# Acute and Chronic Pain Learning and Teaching in Medical School—An Observational Cross-Sectional Study Regarding Preparation and Self-Confidence of Clinical and Pre-Clinical Medical Students

**DOI:** 10.3390/medicina55090533

**Published:** 2019-08-26

**Authors:** Kacper Lechowicz, Igor Karolak, Sylwester Drożdżal, Maciej Żukowski, Aleksandra Szylińska, Monika Białecka, Iwona Rotter, Katarzyna Kotfis

**Affiliations:** 1 Student Science Club at the Department of Anesthesiology, Intensive Therapy and Acute Intoxications, Pomeranian Medical University, 70-111 Szczecin, Poland; 2 Department of Anesthesiology, Intensive Therapy and Acute Intoxications, Pomeranian Medical University, 70-111 Szczecin, Poland; 3 Department of Medical Rehabilitation and Clinical Physiotherapy, Pomeranian Medical University, 71-210 Szczecin, Poland; 4 Department of Pharmacokinetics and Therapeutic Drug Monitoring, Pomeranian Medical University, 70-111 Szczecin, Poland

**Keywords:** pain, medical education, student, pain scales, BPS, CPOT, delirium

## Abstract

*Background and objectives:* Adequate pain management is a major challenge of public health. The majority of students graduating from medical schools has insufficient education and experience with patients suffering pain. Not enough is being taught regarding pain in non-verbal patients (children, critically ill in the intensive care unit, demented). Chronic pain is the most difficult to optimize and requires appropriate preparation at the level of medical school. Our aim was to evaluate attitudes, expectations and the actual knowledge of medical students at different levels of their career path regarding the assessment and treatment of acute and chronic pain. *Materials and Methods:* We performed an observational cross-sectional study that was based on a survey distributed among medical students of pre-clinical and post-clinical years at the Pomeranian Medical University in Szczecin, Poland. The survey included: demographic data, number of hours of formal pain teaching, actual knowledge of pain assessment, and pain treatment options in adults and children. *Results:* We received responses from 77/364 (21.15%) students and 79.2% of them rated the need to obtain knowledge regarding pain as very important (10/10 points). Post-clinical group declared having on average 11.51 h of acute pain teaching as compared to the 7.4 h reported by the pre-clinical group (*p* = 0.012). Graduating students also reported having significantly more classes regarding the treatment of chronic pain (6.08 h vs. 3.79 h, *p* = 0.007). The average level of comfort in the post-clinical group regarding treatment of acute pain was higher than in the pre-clinical group (6.05 vs. 4.26, *p* = 0.006), similarly with chronic pain treatment in adults (4.33 vs. 2.97, *p* = 0.021) and with pain treatment in children (3.14 vs. 1.97, *p* = 0.026). *Conclusions:* This study shows that education about pain management is a priority to medical students. Despite this, there continues to be a discrepancy between students’ expectations and the actual teaching and knowledge regarding effective pain management, including the vulnerable groups: chronic pain patients, children, and critically ill people.

## 1. Introduction

One of the most important challenges of public health is adequate pain management and the problem of under-treatment of pain [1]. It has been estimated that up to 30% of patients suffer pain worldwide [2,3]. The most common patient complaint in the emergency department is acute pain for both adults and children [4,5]. A recent study has shown that 40% of visits, in general, practice is caused by pain and half of this group suffers from chronic pain lasting over six months. In addition, only up to 15% of the population reports to their family physician with chronic pain, so the rest of patient suffer without help [6,7]. Acute and chronic pain can both lead to serious consequences, including prolonged hospital stay, increased healthcare costs, sleep disturbance, and depression [8]. It has been shown that pain in non-verbal patients (critically ill, with cognitive disorders) remains undertreated and requires specific behavioral tests for diagnosis; otherwise, it leads to agitation and delirium with all its consequences [9,10,11,12].

There seems to be a gap between the advances in pain management and the actual application of this knowledge in routine clinical practice. Acute, chronic, and cancer pain remains unsuccessfully controlled, which is partly due to the lack of knowledge of students and later doctors [13]. Those who have not been properly trained in the field of pain medicine may not be able to recognize, correctly diagnose, or treat pain [14]. According to the available literature, the attitude towards treatment of chronic non-cancer pain by doctors begins early in the medical school [15]. Previous publications support the view that medical students are concerned about the treatment of patients with chronic pain [16]. Studies have shown that most medical students have insufficient experience and education with patients suffering from pain, and that chronic pain is the most difficult to optimize and it requires appropriate preparation at the level of medical schools [17,18]. The inability to teach medical students and transfer skills in the treatment of chronic pain is consistent with the statement that pre-clinical programs are not well coordinated with clinical programs [19]. Giordano and Boswell have clearly stated that the mechanisms of pain and analgesia are taught during the basic courses of neurobiology and pharmacology, but this is not directly related to the complexity and clinical approach for the patient with chronic pain [20].

The use of opioids remains as one of the biggest problems among young doctors. This may be related to a conviction that prescribing narcotic drugs, including opioids, might have legal consequences [21]. Students of medical schools are not adequately educated in the proper dosage and use of these medicines. Reasons are found both in the incorrectly distributed curriculum and in patterns learned from older colleagues [14,22]. When planning treatment, not only the nature of pain should be taken into account, but also the age of the patient. Treatment of children requires dosage that is related not only to age, but also to their body weight. This can often cause problems for young inexperienced doctors who avoid the administration of this group of medicines in emergency departments [4,5].

There are several hypotheses explaining this phenomenon, including the doctor’s lack of comfort in terms of drug dosage and side effects [23]. In addition, there is a general belief that patients at the extremes of age (i.e., young children) do not feel pain like adults and they will not remember it. It should be noted that, in most curricula regarding pain in medical schools, there is a greater emphasis on pain management in adults, hence ignorance regarding the understanding of pain treatment and assessment in children [24,25,26]. There is also a belief in the inability of young children and patients who cannot communicate to verbalize their needs. This includes mechanically ventilated and sedated patients in the intensive care unit and patients with dementia [9,10,27]. In addition, instead of providing a separate educational block, a one-subject pain education program is often divided in medical schools, and this subject is taught as small elements that are spread over many basic subjects. It triggers discomfort in the assessment of pain mainly in younger patients and the lack of proper treatment by physicians [28].

The achievement of the correct diagnosis and effective treatment of pain itself is very complicated, as it requires the doctor’s holistic knowledge not only in the field of pharmacology but also in many other medical subjects. It also requires cooperation between different specialists dealing with pain, including pain specialist, anesthesiologist, neurologist, neurosurgeon, pharmacist, psychologist, and physiotherapist that should provide coordinated care to the patient. The curriculum at the Pomeranian Medical University in Szczecin (Poland), as in many other medical schools worldwide, does not provide a separate coordinated faculty for pain treatment. Knowledge regarding this subject is dispersed in many preclinical and clinical activities, such as pharmacology, anesthesiology and intensive care, palliative medicine, and pediatrics. The majority of these courses are focused on the treatment of acute pain and this should be regarded as a reason why chronic pain is underdiagnosed and inadequately treated.

The aim of this study was to evaluate the attitudes and actual knowledge of medical students at different levels of their career path (pre-clinical and post-clinical years) regarding the assessment and treatment of acute and chronic pain. Not enough is being taught regarding pain in non-verbal critically ill patients in the intensive care unit and this is the first study addressing this problem.

## 2. Materials and Methods

### 2.1. Study Group and Data Collection

All medical students after year IV (pre-clinical, after pharmacology) and after year VI (post-clinical) from the Faculty of Medicine at the Pomeranian Medical University in Szczecin (Poland) were identified through the student’s administration office and then invited to complete an online survey on a dedicated platform. The questionnaires were accessed anonymously after logging-in and available to students over the last three weeks of their educational activities in June 2019 (1–21 June 2019). The respondents were invited with an opening letter (1 June 2019) and reminders were sent out at weekly intervals thereafter (8th and 15th of June). The students were divided into two groups—Group I (pre-clinical) after year IV and Group II (post-clinical) after year VI.

### 2.2. Survey Development

A dedicated survey was created that consisted of open and closed format questions according to guidelines for survey development to perform this study. A team, including an anesthesiologist and intensivist (KK), neurologist (IR), statistician (AS), and a medical student (KL) created a preliminary questionnaire. Questionnaire items were identified through literature review. The survey was consulted by senior experts in research and in clinical medicine, which are involved in pain teaching and treatment, namely an anesthesiologist, pain specialist, neurologist, pharmacologist, and physiotherapist. The pilot survey was presented to three residents and three medical students not involved in the study to evaluate the quality of the questionnaire and to ensure its content validity and phrasing clarity.

The survey was structured into sections:

- Questions regarding demographic data (e.g., sex, age, year of study);
- Recall and opinion of the students regarding pain teaching;
- Knowledge about scales available for pain assessment;
- Knowledge regarding common pain medication dosing; and,
- Questions regarding facts and myths in the treatment of acute and chronic pain.

The questions included in the final version of the survey were single and multiple choice, those regarding preference were based on a 10-point numerical scale (0–10 points). Questions regarding knowledge concerned the use of the most popular medical compounds (paracetamol, ibuprofen, morphine) among adults and children. The statements regarding facts and myths were stratified into a five-point Likert scale (0—strongly disagree to 5—strongly agree). Before disseminating the questionnaire, the following elements were evaluated by senior authors: layout, format, question order, and appropriate vocabulary. All questions and the distractors were evaluated as sensible and easy to understand for someone with basic medical knowledge.

### 2.3. Ethical Consideration

This study was an anonymous survey and as such was an exempt from the approval of the ethics committee. The study received an official written waiver from the Bioethics Committee of the Pomeranian Medical University in Szczecin, no. KB-0012/168/06/19 issued on 5 June 2019. The University authorities were informed about the study.

### 2.4. Statistical Analysis

The data are presented as means and standard deviations for continuous variables and numbers and percentages for categorical variables. Chi-square test or Chi-square test with Yates correction was used to compare the qualitative data between the two groups of patients. Mann–Whitney U test was used to compare continuous variables. *p*-value was considered significant when ≤0.05. All analyses were performed by an experienced biostatistician using licensed software Statistica 13 (StatSoft, Inc. Tulsa, OK, USA).

## 3. Results

### Baseline Characteristics

We received responses to the survey from 77/364 students (21.15%), with 34/166 (20.5%) answers from pre-clinical students and 43/198 (21.7%) from the post-clinical group (Table 1). In Group I, the majority of students were 19–25 years old (94.12%) and in Group II the students were statistically significantly older, as 62.79% of the respondents were between 25 and 30 years of age. In the pre-clinical group, women accounted for 61.76% and in the post-clinical group 65.12%. In the month preceding the completion of the survey, the use of pain medications among respondents and/or their relatives was 20.59% (regularly) and 32.35% (sporadically) in the pre-clinical group. In the group of graduating students, 6.98% respondents or their close relatives regularly used pain medications, whereas 27.91% used them sporadically. In both groups, 61/364 (79.22%) of students assessed the need to obtain knowledge about pain as very important, appointing 10 out of 10 points, as seen in Table 1.

The students also answered the question about the number of hours in the curriculum regarding pain management that they recall from their experience (Table 2). The post-clinical group declared having on average 11.51 h of acute pain teaching as compared to the 7.4 h reported by the pre-clinical group (*p* = 0.012). Graduating students also reported having significantly more classes regarding the treatment of chronic pain 6.08 h vs. 3.79 h (*p* = 0.007).

The graduates reported more bedside classes than younger colleagues, however these values were not statistically significant in the treatment of adults and children. Both pre- and post-clinical group considered seminars in small groups as the best way to learn (70.59% vs. 67.44%, *p* = 0.654), with bedside classes as their second choice (29.41% vs. 27.91%, *p* = 0.654). Interestingly, when asked whether the differences between chronic and acute pain treatment have been explained, more students in Group I stated that they have been given enough information on the topic (32.35% vs. 27.91%, *p* = 0.364).

As it may be seen in Table 3, the source of knowledge about pain treatment is significantly different for both groups (*p* = 0.009). Post-clinical students were much more likely to indicate palliative medicine as the main source of knowledge regarding pain management (32.56% vs. 2.94%), contrary to the preclinical students, who based their knowledge almost entirely on pharmacology (88.24% vs. 58.14%). This is due to the fact that palliative medicine is a subject taught during clinical years.

To assess the knowledge regarding pain, students were asked about the elements of the World Health Organization (WHO) analgesic ladder, pain assessment scales, and knowledge of non-pharmacological methods of pain management (Table 4). Post-clinical students significantly more often admitted recalling the NRS and VAS scale (*p* = 0.003 and *p* = 0.004). Knowledge regarding behavioral pain scales used in the ICU was minimal, as reported by 3/77 (3.89%) student regarding BPS and 3/77 (3.89%) students regarding CPOT. The overall number of known scales was also higher in Group II (*p* = 0.032). Knowledge of non-pharmacological methods of pain management was similar in the majority of respondents. Students in Group II compared to Group I more often mentioned peripheral and central blockades (83.72% vs. 44.12%, *p* < 0.001) and physical therapy and rehabilitation (79.07 vs. 52.94%, *p* = 0.029) as a treatment option for pain. When asked about the WHO analgesic ladder (i.e., to indicate the correct answers to appoint medications on level II of the ladder), students from Group I more often gave correct answers (26.47% vs. 11.63%, *p* = 0.168).

**Figure 1:**
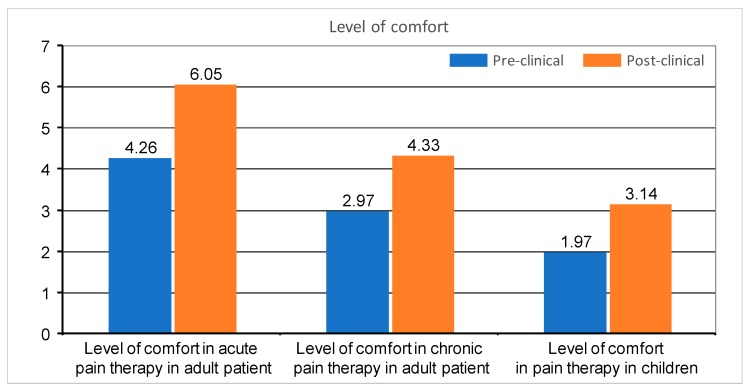
Level of comfort of pre-clinical and post-clinical students regarding treatment of acute pain in adults (*p* = 0.006), chronic pain in adults (*p* = 0.021) and pain in children (*p* = 0.026). Comfort is shown as mean for the whole group (on a 0–10 scale) and correct dosing as percentage of student choosing a correct dose (0–100%).

On a similar linear 11-point scale, where 0 was marked as complete lack of comfort and 10 meant feeling very comfortably, the students were asked about their level of comfort in the treatment of acute and chronic pain in adults and the treatment of pain in children. The average level of comfort in the post-clinical group regarding the treatment of acute pain was significantly higher than in the pre-clinical group (6.05 vs. 4.26, *p* = 0.006), similarly with chronic pain treatment in adults (4.33 vs. 2.97, *p* = 0.021) and pain treatment in children (3.14 vs. 1.97, *p* = 0.026). Both groups were much more confident in the treatment of acute pain in adults, followed by chronic pain treatment in adults, while the least confident in the treatment of pain in children (Figure 1).

**Figure 2:**
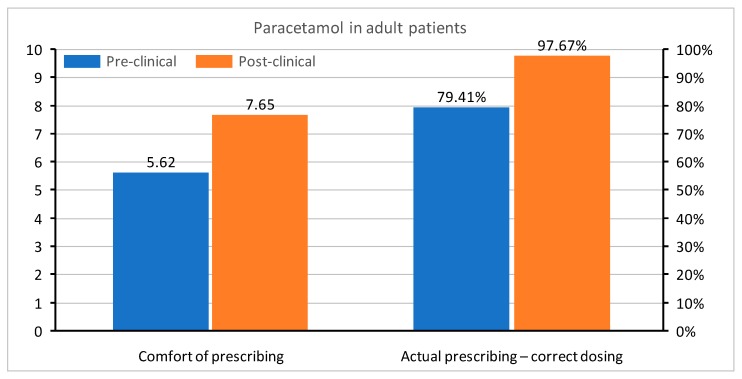
Comfort of prescribing (*p* < 0.001) and the actual prescribing of correct doses of paracetamol in adult patients (*p* = 0.025) in pre-clinical and post-clinical students [comfort is shown as mean for the whole group (on a 0–10 scale) and correct dosing as percentage of student choosing a correct dose (0–100%)].

**Figure 3:**
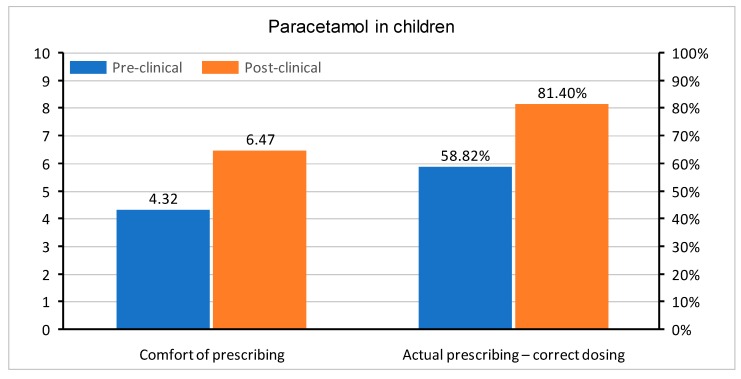
Comfort of prescribing (*p* = 0.001) and the actual prescribing of correct doses of paracetamol in pediatric patients (*p* = 0.054) in pre-clinical and post-clinical students [comfort is shown as mean for the whole group (on a 0–10 scale) and correct dosing as percentage of student choosing a correct dose (0–100%)].

**Figure 4:**
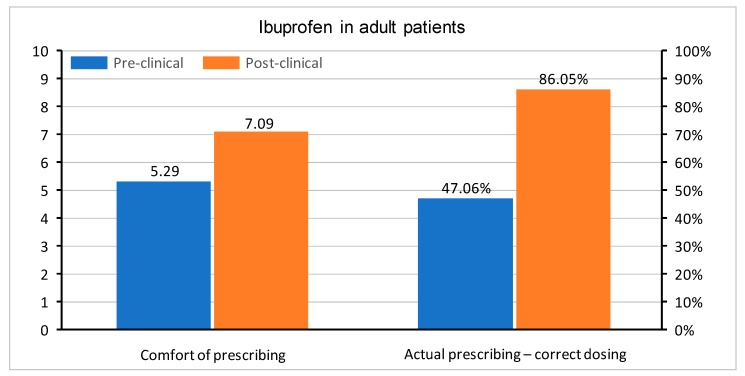
Comfort of prescribing (*p* = 0.008) and the actual prescribing of correct doses of oral ibuprofen in adult patients (*p* < 0.001) in pre-clinical and post-clinical students [comfort is shown as mean for the whole group (on a 0–10 scale) and correct dosing as percentage of student choosing a correct dose (0–100%)].

**Figure 5:**
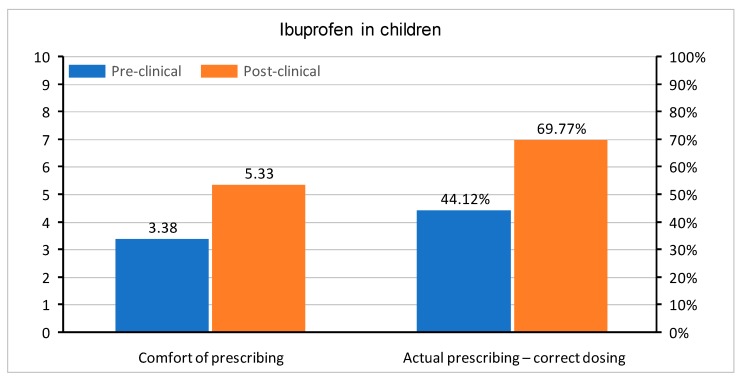
Comfort of prescribing (*p* = 0.002) and the actual prescribing of correct doses of oral ibuprofen in pediatric patients (*p* = 0.023) in pre-clinical and post-clinical students [comfort is shown as mean for the whole group (on a 0–10 scale) and correct dosing as percentage of student choosing a correct dose (0–100%)].

**Figure 6:**
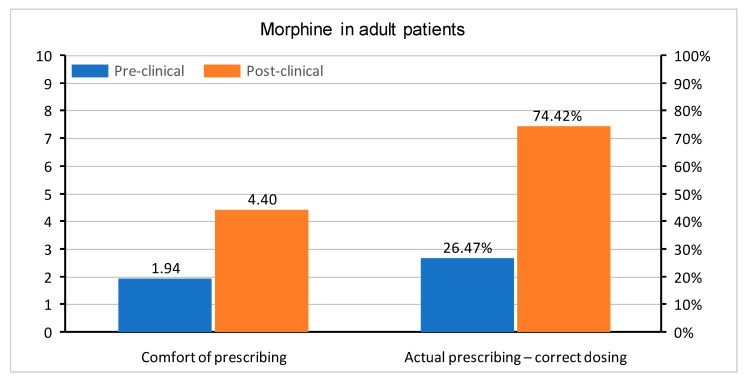
Comfort of prescribing (*p* < 0.001) and the actual prescribing of correct doses of intravenous morphine in adult patients (*p* < 0.001) in pre-clinical and post-clinical students [comfort is shown as mean for the whole group (on a 0–10 scale) and correct dosing as percentage of student choosing a correct dose (0–100%)].

**Figure 7:**
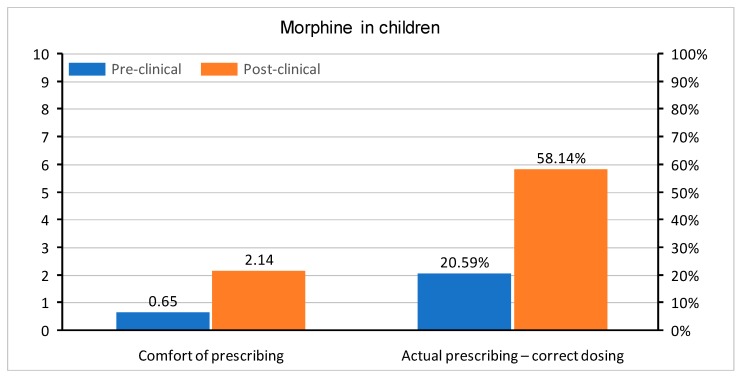
Comfort of prescribing (*p* < 0.001) and the actual prescribing of correct doses of intravenous morphine in pediatric patients (*p* = 0.002) in pre-clinical and post-clinical students [comfort is shown as mean for the whole group (on a 0–10 scale) and correct dosing as percentage of student choosing a correct dose (0–100%)].

In questions regarding medication prescription, the correct single dose of paracetamol was set to be between 500 and 1000 mg, ibuprofen between 200 and 400 mg, and morphine 0.1–0.15 mg/kg. Doses in children were determined, as follows: for paracetamol 10–20 mg/kg, ibuprofen 5–10 mg/kg, and morphine 0.1–0.2 mg/kg. All of the doses were based on the manufacturer’s product characteristics of medications registered in Poland. The questions were asked about a healthy 75 kg adult male and a healthy 10 kg child without any co-morbidities. Apart from the actual knowledge regarding drug dosing, the students were also asked about their level of comfort when prescribing paracetamol, ibuprofen, and morphine in adults and children. In all questions, the graduates were much more confident with each drug. A comparison of comfort in the prescription of drugs and correct medication doses in both groups is shown in Figure 2, Figure 3, Figure 4, Figure 5, Figure 6 and Figure 7. Self-evaluation of the respondents was usually lower than the actual knowledge tested as prescribing the correct dose of a medication in question.

Figure 6 and Figure 7 show responses regarding the comfort of prescribing of intravenous morphine and the actual correct dosage of this medication. The actual knowledge in post-clinical group was higher than the comfort with which the respondents approached the issue, both when prescribing intravenous morphine in adults (correct dosing 74.42% of Group II, comfort—mean 4.4) and in children (correct dosing 58.14% of Group II, comfort—mean 2.14).

The survey also included questions regarding facts and myths regarding pain management. Most of the questions received similar answers between the two groups, however post-clinical students significantly more often claimed that pain treatment does not mask the cause of pain and reduces the accuracy of the diagnosis. Pre-clinical students more often claimed that it is easier to become addicted to opioids in chronic than in acute pain. Table 5 shows complete results.

The students were also asked about the potential elements that influence the treatment of pain (Table 6). The most important identified barrier to treatment of pain in the pre-clinical group was “Side-effects of medications and contraindications”, followed by “Patient co-morbidities and history of chronic pain” and “Fear of drug addiction”. Post-clinical students significantly more often pointed to “Insufficient physician training” (39.53% vs. 14.71%, *p* = 0.032). Good communication between the doctor and the patient was considered to be the most important facilitating factor in pain management in both groups. The factor that was reported to facilitate the treatment of chronic pain in the pre-clinical group was “Good patient–physician communication”, whereas it was “Physician education” in the post-clinical group.

## 4. Discussion

The results of our study have shown that students immediately prior to graduation do not feel comfortable regarding pain treatment and their knowledge regarding the dosing of analgesic medications is insufficient. The majority of the respondents in the post-clinical group, i.e., students who have already completed their curriculum in medicine and are to become medical doctors, regarded pain treatment as a priority and graded it as having 10/10 importance level. As expected, the knowledge regarding drug dosing was more accurate in this group. They prescribed correct doses of paracetamol, ibuprofen, and morphine for adults and children (*p* < 0.05) more frequently than their younger colleagues. New knowledge coming from our study is the important piece of information regarding pain in the critically ill patients and issues that are associated with chronic pain assessment and treatment. In our study, as the biggest barrier hindering the treatment of pain in adults, the students of the pre-clinical group recognized the side effects of the drugs and their contraindications, followed by co-morbidities and the history of chronic pain, as well as the fear of addiction. Students from the post-clinical group significantly more often pointed to insufficient training of the doctor (39.53% vs. 14.71%, *p* = 0.032) as the reason for uncertainty in the decisions made during treatment.

A survey of recent graduates of medicine in Ireland showed that there was a discrepancy between the confidence in prescribing and the actual accuracy of prescription of medicines. The students reported that 89% of them feel confident in writing prescriptions, but only 58% indicated correct drug dose calculations, and only 28% agreed that their medical education prepared them for prescribing medicines [29]. Dosage accuracy and form of administration should be important aspects of the pain management study program.

In the group of our subjects, one can notice how significantly the source of knowledge regarding pain treatment changes for both groups. Post-clinical students were much more likely to point to palliative medicine as the main source of knowledge about pain management (32.56% vs. 2.94%), as opposed to preclinical years where students base their knowledge on pharmacology (88.24% vs. 58.14%). In addition, they more often gave correct answers to most questions. The students from the Post-clinical group had more experience and were able to translate their theoretical knowledge into a doctor-patient relationship. In a study that was performed by Egnew et al., the experiences of students and academic staff on how students learn the skills of the doctor-patient relationship in their clinical medical education were recorded and analyzed to assess how this translates into their self-confidence in practice [19]. The respondents indicated that the pre-clinical literacy programs were not well coordinated with clinical programs. As part of the clinical teaching program, respondents perceived a discrepancy between general practice and theoretical science. Respondents recommended greater concentration and assessment of the interpersonal skills of students in a clinical setting.

Another important remark that was made by lecturers was the tension between service obligations and teaching students in hospital attachments that contributed to insufficient focus on communication and acquisition of skills related to relationships, and did not strengthen teaching in clinical, preclinical, and outpatient settings. Teaching the skills of the doctor-patient relationship can be strengthened by coordinating pre-clinical and clinical pain teaching programs and by requiring observation and structured feedback associated with clear criteria for acquiring students’ skills in all clinical learning experiences [19,20].

In our group of surveyed students, both in the pre-clinical group and in the senior students group, the minimal number of hours of pain treatment (5 h) was higher than in some medical schools in Europe and North America, where this time has not been reached. Mezei et al., in their study performed in year 2011, found that many American medical schools did not report any didactic hours regarding pain, and many of them required only five or fewer hours of such education. Electives were available in only 16% of schools, and 80% of American medical schools did not have such classes in pain. In addition, only 4% of US medical schools reported access to courses on integrated pain management [28].

According to the International Association on the Study of Pain, in Germany the subject is taught within a 15 h-course during the fifth year of medical school [30]. In an article from 2014, Kopf et al. described the introduction of a formal compulsory curriculum in medical schools in Germany [31]. It was introduced in 2012 and it was to serve as a potential solution for training future doctors with knowledge and comfort in the treatment of acute pain. Awareness of this important issue should increase the knowledge and competence in biopsychosocial measurements of pain and risk assessment, improve understanding of persistent pain as a chronic complex condition, and expand the role of interdisciplinary treatment [30].

There are several factors that may have influenced the outcome of this study. The relatively small study sample may have added to the underreporting of problems that are associated with pain education. Moreover, having no official “pain rotation” or “pain subject” could have increased the uncertainty of the respondents regarding the actual hours spent in pain-related classes. In the course of studies at the Pomeranian Medical University, there are no classes or faculties that are solely dedicated to the treatment of pain. Students learn about pain treatment during pharmacology (10 h) and palliative medicine (6 h). During pharmacology, they learn about drug groups and classes: nonsteroidal anti-inflammatory drugs (NSAIDs), nonopioids, opioids, local anesthetics, and drugs for neuropathic pain. The 3 h of palliative medicine are intended for the treatment of chronic pain. All other information regarding pain, including pain in critically ill non-verbal patients, is provided during bedside teaching and does not follow any formal structure. At the moment there are no official guidelines regarding pain curriculum for medical schools in Poland. A statement from the Polish Association for the Study of Pain would have been a major help for medical school authorities. A call for change should be issued regarding this important problem.

In year 2014, a publication describing the teaching of pain in Europe appeared—the results of the APPEAL (Advancing the Provision of Pain Education and Learning) study were published. In their analysis, Briggs et al. included information regarding 242 Medical Schools from 15 European countries [32]. In this respect, France came out the best, where 87% of universities had a dedicated pain therapy module. In Poland, 11 universities took part in this study. None of them reported having a separate module and they all declared teaching regarding pain during other activities and 100% of them were obligatory [31]. This study is consistent with the data that were obtained among our students. The source of knowledge declared by respondents of our survey is classes where the pain was only mentioned (palliative medicine, pharmacology). When considering that about 80% of students declare the need to obtain such knowledge as indispensable (82.35% pre-clinical vs. 76.74 post-clinical students), it would be appropriate to consider introducing rotations that are dedicated to pain therapy, as seen in other countries.

Both groups of students felt much more confident in the treatment of acute pain in adults. The respondents felt least confident in relation to the treatment of pain in children. According to the medical literature, the students feel uncomfortable both in dealing with pain in children and in its assessment. Ameringer et al. showed poor teaching results and negligible time (1 h) in coping with pain and its assessment in children [33]. More importantly, this study showed improvement through interventions, such as internet modules and learning that is based on clinical problems. Interestingly, they showed that confidence in the assessment of childhood pain improved from only 6% to 25% after using the online module. As many as 71% of students said they intend to introduce positive changes in their practice based on a training module in the field of pain therapy [32].

In year 2015, Tran et al. performed a study involving students of the University of Alberta, Canada [23]. Clerkship students prescribed incorrect doses of pain medications in more than 50% of cases. Students of lower years (Pre-clerkship) indicated lectures as the best source of knowledge, while senior seminaries in small groups and exercises at the patient’s bed. Students of the Pomeranian Medical University in both the younger and the older group pointed to the seminars in small groups as the best source of knowledge. When comparing the correct values of the prescribed drugs, we can see that older students in Poland achieved better results in prescribing paracetamol to adults (97.67% vs. 45.8%). It can lead to a conclusion that exercises in small groups bring better results than lectures. However, it should be remembered that, in both studies, different criteria of correct doses have been adopted, which may have affected the results achieved [23].

### 4.1. Study limitations and Strengths

Our study is not without limitations. First, the fact that this is a single-center study limits its generalizability. Second, the sample size is relatively small. The response rate was relatively low at 20.3%, yet acceptable, as compared with other questionnaire based on-line surveys. Despite formal reminders, only one-fifth of the students responded to the survey. The similar problem has been reported by other authors who performed surveys among medical school students [23,34,35]. The response rate could have been improved by a different timing of the study. We distributed the questionnaire during the last month of academic activities to get the most comprehensive picture of the situation. However, during that time, many students from the non-respondent group could have been involved in final exams, rotations or simply were already on vacation. Third, when constructing the survey, we could have omitted some barriers and facilitators we were not fully aware of, i.e., there are more myths/facts to the subject of pain treatment, but the limited length of the survey had to be taken into account. Moreover, the recall bias is another potential limitation of this study. We also believe that only including medical students is a potential limitation. We could have invited nursing, physiotherapy, psychology, or public health students, although the survey tool would need amendments to accommodate these groups. We plan to do that in near future.

Nevertheless, this study adds an important piece of information regarding pain teaching in medical schools, especially highlighting the need to increase the emphasis on chronic pain and pain in the critically ill patients. The Declaration of Montreal that was issued by International Association on the Study of Pain (IASP) in year 2010 stated that: “All people have the right to have access to appropriate assessment and treatment of pain by adequately trained health-care professionals.” Also, in Poland, in March 2017, the Patient’s Right Act was enriched with paragraph 20a stating that “Every patient has the right to pain treatment”. Despite the above-mentioned declarations and statements, there continues to be a gap between research and knowledge regarding effective pain management and the real-life delivery of adequate and appropriate patient care. Taking the novelty in legislation into account, there is a serious need to adjust medical university curriculum for all future healthcare professionals.

In year 2013, the European Pain Federation (EFIC) issued a document called “The Pain Management Core Curriculum for European Medical Schools” (first issued in year 2008) that encourages an interdisciplinary teaching approach to aid the construction of pain curriculum and encourages students to concentrate on the most frequent pain syndromes and the most common treatment options (https://europeanpainfederation.org). Some authors have proposed an approach based on partnership between the medical faculty and medical students [36]. The aim of Bradshaw et al. was to reshape the medical education about pain and create a curriculum that involves empathy and care-based ′student-led learning′ management competencies [37,38]. In their initiative, Bradshaw et al. decided to collaborate with students to apply a series of predesigned steps to modify the multiyear presentation of pain education into a coordinated rotation [37]. The curriculum was reorganized to present pain as a disease state and a common public health burden, not just a symptom, to meet the graduating students’ needs to gain competency and propose a broader social perspective on pain [38]. The working group created a preclinical curriculum inventory and a fourth-year month-long elective rotation with a dedicated reading list and practical classes in out-of-university pain clinics, seminars, and discussions with pain mentors that coordinated basic knowledge with clinical observation.

### 4.2. Future Directions

The authors believe that the results of this study might be relevant to the subject of pain-related curriculum in other medical schools. Below is an outline of a comprehensive plan for improvement that may be used in other medical universities in the future:

1. medical school curriculum should include a separate compulsory subject dedicated to pain diagnosis and treatment and involve new teaching techniques;
2. a dedicated medical textbook should be provided including not only the pathophysiology of pain and pharmacology, but also specific diagnostic tests and treatment schemes including children, chronic pain, pain in critically ill patients in the ICU, pain in elderly and in dementia, as well as exercises based on clinical cases;
3. both the curriculum and textbook should be country specific, including local medications and regulations, as well guidelines on the conversion of doses of different drugs;
4. the subject should be based on a partnership between the faculty and the students and be composed of two parts—first part to be introduced at the very beginning of clinical courses, but after a course in pharmacology and the second part during the clinical years. It should be based on seminars and exercises in small groups, both at patients’ bedside and in outpatient pain clinics;
5. the subject should involve specialists in different fields: pain specialist, anesthesiologist, intensivist, neurologist, neurosurgeon, pharmacologist, psychologist and physiotherapist; and,
6. we suggest that additional four to six teaching hours should be devoted to pain treatment at a Medical Simulation Centre where students of the preclinical years could translate newly acquired knowledge into practice in arranged scenarios.

## 5. Conclusions

This study shows that medical students regard education about pain management as a priority. Despite this declared need there continues to be a discrepancy between students’ expectations and the actual knowledge about effective acute and chronic pain management and the amount of time regarding pain treatment in the medical school curriculum. Additional emphasis should be placed on teaching effective pain management in the most vulnerable groups of patients: children, critically ill, and those suffering from chronic pain. All future medical school curricula should follow this trend to meet the needs of medical students and the society.

## Figures and Tables

**Table 1:** Baseline characteristics of the study group.

Variables	Total (*n =* 77)	Group I Pre-Clinical (*n =* 34)	Group II Post-Clinical (*n =* 43)	*p*-Value
Total number of students invited to participate in the study, *n*	364	166	198	--
Responses to the survey, *n* (%)	77/364 (21.15)	34/166 (20.5)	43/198 (21.7)	--
**Demographic data**				
Gender [female], *n* (%)	49/77 (63.63)	21 (61.76)	28 (65.12)	0.761
Age [19–25 years], *n* (%)	48 (62.34)	32 (94.12)	16 (37.21)	<0.001
Age [25–30 years], *n* (%)	29 (37.66)	2 (5.88)	27 (62.79)	
**Personal experience**				
Sporadic use of analgesic medications *, *n* (%)	23 (29.87)	11 (32.35)	12 (27.91)	0.141
Regular use of analgesic medications *, *n* (%)	10 (12.98)	7 (20.59)	3 (6.98)	
**Personal value of knowledge about pain treatment ^**				
7 points, *n* (%)	2 (2.59)	2 (5.88)	0 (0.00)	0.162
8 points, *n* (%)	7 (9.09)	1 (2.94)	6 (13.95)	
9 points, *n* (%)	7 (9.09)	3 (8.82)	4 (9.30)	
10 points, *n* (%)	61 (79.22)	28 (82.35)	33 (76.74)	

Legend: *n*—number of respondents, * by the respondent or respondent’s family for more than one week during the month preceding the survey, ^ Scale 1–10.

**Table 2:** Recall of formal pain teaching during medical school.

Variables	Group I Pre-Clinical (*n =* 34)	Group II Post-Clinical (*n =* 43)	*p*-Value
Hours of acute pain education reported, mean ± SD	7.40 ± 4.00	11.51 ± 8.78	0.012
Hours of chronic pain education reported, mean ± SD	3.79 ± 3.22	6.08 ± 4.63	0.007
Type of classes about pain treatment in adults—reported			
Seminars in small groups, *n* (%)	28 (82.35)	33 (76.74)	0.786
Lectures, *n* (%)	5 (14.71)	8 (18.60)	
Bedside teaching, *n* (%)	0 (0.00)	1 (2.33)	
All mentioned above, *n* (%)	1 (2.94)	1 (2.33)	
Type of classes about pain treatment in children—reported			
Seminars in small groups, *n* (%)	18 (52.94)	14 (32.56)	0.066
Lectures, *n* (%)	5 (14.71)	11 (25.58)	
Bedside teaching, *n* (%)	0 (0.00)	6 (13.95)	
All mentioned above, *n* (%)	0 (0.00)	1 (2.33)	
None of the above, *n* (%)	11 (32.35)	11 (25.58)	
Type of classes about pain treatment—preferred			
Seminars in small groups, *n* (%)	24 (70.59)	29 (67.44)	0.654
Lectures, *n* (%)	10 (29.41)	12 (27.91)	
Bedside teaching, *n* (%)	0 (0.00)	1 (2.33)	
Seminars and bedside classes, *n* (%)	0 (0.00)	1 (2.33)	
Differences between acute and chronic pain treatment			
The problem was inadequately discussed, *n* (%)	20 (58.82)	30 (69.77)	
The problem was adequately discussed, *n* (%)	11 (32.35)	12 (27.91)	0.364
The problem was not discussed at all, *n* (%)	3 (8.82)	1 (2.33)	

Legend: *n*—number of respondents, SD—standard deviation.

**Table 3:** Courses in medical school indicated as providing knowledge about pain treatment.

Variables	Group I Pre-Clinical (*n =* 34)	Group II Post-Clinical (*n =* 43)	*p*-Value
Palliative medicine, *n* (%)	1 (2.94)	14 (32.56)	0.009
Pharmacology, *n* (%)	30 (88.24)	25 (58.14)	
Emergency medicine, *n* (%)	0 (0.00)	2 (4.65)	
Anaesthesiology and intensive care, *n* (%)	1 (2.94)	2 (4.65)	
Geriatrics, *n* (%)	1 (2.94)	0 (0.00)	
Student scientific circle of interest, *n* (%)	1 (2.94)	0 (0.00)	

Legend: *n*—number of respondents.

**Table 4:** Knowledge regarding pain assessment and treatment among medical students.

	Group I Pre-Clinical (*n =* 34)	Group II Post-Clinical (*n =* 43)	*p*-Value
Knowledge regarding pain assessment scales			
NRS, *n* (%)	8 (23.53)	26 (60.47)	0.003
VAS, *n* (%)	10 (29.41)	28 (65.12)	0.004
FPS, *n* (%)	20 (58.82)	25 (58.14)	0.952
FLACC, *n* (%)	3 (8.82)	3 (6.98)	0.898
BPS, *n* (%)	1 (2.94)	2 (4.65)	0.835
CPOT, *n* (%)	0 (0.00)	3 (6.98)	0.116
Knowledge regarding pain treatment options (other than pharmacotherapy)			
Regional and central blocks, *n* (%)	15 (44.12)	36 (83.72)	<0.001
Surgical treatment, *n* (%)	14 (41.18)	21 (48.84)	0.503
Thermolesion, *n* (%)	0 (0.00)	1 (2.33)	0.906
Neuromodulation, *n* (%)	5 (14.71)	9 (20.93)	0.685
Acupuncture, *n* (%)	7 (20.59)	9 (20.93)	0.806
Rehabilitation and physical therapy, *n* (%)	18 (52.94)	34 (79.07)	0.029
Psychological treatment, *n* (%)	17 (50.00)	22 (51.16)	0.898
Correct answer about WHO analgesic ladder			
Correct answer regarding level II of the analgesic ladder, %	26.47	11.63	0.168

Legend: *n*—number of respondents, WHO—World Health Organization, NRS—Numerical Rating Scale, VAS—Visual Analogue Scale, FPS—Faces Pain Scale, FLACC—The Face, Legs, Activity, Cry, Consolability scale, BPS—Behavioral Pain Scale, CPOT—Critical Care Pain Observation Tool.

**Table 5:** Students’ opinions regarding pain-related facts and myths.

Variables	Definitely Disagree *n* (%)	Rather Disagree *n* (%)	No Opinion *n* (%)	Rather Agree *n* (%)	Definitely Agree *n* (%)
**“Risk of addiction is a cause of not prescribing opioids in pain.”**	*p* = 0.248				
Pre-clinical, *n* (%)	1 (2.94)	3 (8.82)	0 (0.00)	14 (41.19)	16 (47.06)
Post-clinical, *n* (%)	4 (9.30)	7 (16.28)	1 (2.33)	20 (46.51)	11 (25.58)
**“Elderly people have greater risk of analgesic drugs overdose.”**	*p* = 0.194				
Pre-clinical, *n* (%)	2 (5.88)	1 (2.94)	1 (2.94)	13 (38.24)	17 (50.00)
Post-clinical, *n* (%)	3 (6.98)	5 (11.63)	3 (6.98)	21 (48.84)	11 (25.58)
**“The cut-off for initiating pain treatment is NRS > 4/10.”**	*p* = 0.148				
Pre-clinical, *n* (%)	2 (5.88)	3 (8.82)	10 (29.41)	11 (32.35)	8 (23.53)
Post-clinical, *n* (%)	3 (6.98)	2 (4.65)	4 (9.30)	16 (37.21)	18 (41.86)
**“Analgesic drugs mask the pain cause and lower the diagnostic accuracy.”**	*p* = 0.032				
Pre-clinical, *n* (%)	4 (11.76)	11 (32.35)	6 (17.65)	9 (26.47)	4 (11.76)
Post-clinical, *n* (%)	17 (39.53)	14 (67.65)	5 (11.63)	3 (6.98)	4 (9.30)
**“Proper analgesia helps patients to recover quicker after surgery.”**	*p* = 0.068				
Pre-clinical, *n* (%)	1 (2.94)	0 (0.00)	1 (2.94)	13 (38.24)	19 (55.88)
Post-clinical, *n* (%)	4 (9.30)	1 (2.33)	1 (2.33)	5 (11.63)	32 (74.42)
**“Analgesic drugs are the only way to treat pain.”**	*p* = 0.166				
Pre-clinical, *n* (%)	21 (61.76)	9 (26.47)	4 (11.76)	0 (0.00)	0 (0.00)
Post-clinical, *n* (%)	30 (69.77)	8 (18.60)	1 (2.33)	3 (6.98)	1 (2.33)
**“The same analgesic ladder is used in the treatment of chronic and acute pain.”**	*p* = 0.436				
Pre-clinical, *n* (%)	11 (32.35)	11 (32.35)	3 (8.82)	5 (14.71)	4 (11.76)
Post-clinical, *n* (%)	8 (18.60)	13 (30.23)	9 (20.93)	9 (20.93)	4 (9.30)
**“The most popular analgesics in chronic pain treatment are NSAIDs.”**	*p* = 0.650				
Pre-clinical, *n* (%)	10 (29.41)	11 (32.35)	6 (17.65)	4 (11.76)	3 (8.82)
Post-clinical, *n* (%)	10 (23.26)	11 (25.58)	7 (16.28)	11 (25.58)	4 (9.30)
**“Symptoms of chronic and acute pain are the same, only the duration is different.”**	*p* = 0.949				
Pre-clinical, *n* (%)	21 (61.76)	8 (23.53)	1 (2.94)	3 (8.82)	1 (2.94)
Post-clinical, *n* (%)	23 (53.49)	11 (25.58)	2 (4.65)	4 (9.30)	1 (2.33)
**“It is much easier to become addicted to medications in chronic pain, than in acute pain.”**	*p* = 0.037				
Pre-clinical, *n* (%)	1 (2.94)	1 (2.94)	2 (5.88)	7 (20.59)	23 (67.65)
Post-clinical, *n* (%)	4 (9.30)	1 (2.33)	8 (18.60)	16 (37.21)	14 (67.65)
**“Pain scales can measure pain in patients who are unconscious in the ICU.”**	*p* = 0.043				
Pre-clinical, *n* (%)	8 (23.53)	6 (17.65)	14 (41.18)	4 (11.76)	2 (5.88)
Post-clinical, *n* (%)	12 (27.91)	10 (23.65)	5 (11.63)	9 (20.93)	7 (16.28)

Legend: *n*—number of respondents, NRS—Numerical Rating Scale, NSAIDs—Nonsteroidal anti-inflammatory drugs, ICU—Intensive Care Unit.

**Table 6:** Attitudes in pain treatment identified by medical students.

Variables	Group I Pre-Clinical (*n =* 34)	Group II Post-Clinical (*n =* 43)	*p*
**What interferes with optimal pain treatment?**			
Side-effects of medications and contraindications	61.76%	60.47%	0.908
Fear of drug addiction	17.06%	48.84%	0.877
Inadequate training	14.71%	39.53%	0.032
Prejudice of health professionals	17.65%	16.28%	0.883
Patient co-morbidities and history of chronic pain	55.88%	44.19%	0.308
**What facilitates acute pain treatment?**			
Good patient–physician communication	67.65%	83.72%	0.166
Involvement of a multidisciplinary team	50.00%	44.19%	0.612
Good pain re-assessment	44.12%	39.53%	0.685
Previous patient history utilization	29.41%	27.91%	0.913
**What facilitates chronic pain treatment?**			
Physician education	47.06%	55.81%	0.445
Health care team support	41.18%	44.19%	0.791
Good patient–physician communication	52.94%	51.16%	0.877
Use of objective pain scales	38.24%	48.84%	0.352
Involvement of a multidisciplinary treatment/support team	50.00%	51.16%	0.919

Legend: n—number of respondents.
